# Report on the Progress of Anatomy and Physiology

**Published:** 1846-02

**Authors:** W. H. Ranking


### RECORD OF MEDICAL SCIENCE.

### *Report on the Progress of Anatomy and Physiology.*

By W. H. Ranking, M. D.

### § VI.—*Nervous system.*

20. *Functions of the cerebrum and cerebellum.* Dr. Cowan has related two cases of encephaloid carcinoma of the brain, the details of which offer some points of considerable physiological interest. One case is peculiarly instructive, as showing the great amount of cerebral lesion which may exist without being accompanied with any disturbance of the intellectual faculties, and with but slight interruption to the functions of the nervous system generally. The case was chiefly characterized during life by the occurrence of paroxysms or severe pain in the head, which was at first limited to the left side, but eventually became general. These paroxysms were accompanied by severe pain in the right arm and right leg, during the continuance of which the right arm was relaxed and motionless, but regained its power when the pain subsided; this was the only appearance of paralysis noticed; as far as could be ascertained, the sensibility of the arm was not diminished, and the right leg did not participate in the temporary paralysis of motion. There was occasional disturbance of vision in the right eye; and tinnitus of the right ear was a constant symptom; it consisted of a whizzing pulsating noise. There was no appreciable mental disturbance, and the patient walked down stairs the day on which she died. On examining the brain, the greater portion of the medullary substance of the right hemisphere, as also a large portion of that of the left hemisphere, was found converted into a red pulpy mass, presenting all the characters of ordinary encephaloid carcinoma. The grey portion was perfectly healthy to all appearance, which seems to support the favoured doctrine that it is the seat of the intellectual powers. Dr. Cowan suggests that the constant tinnitus may be explained by considering it to have been dependent on the pulsations of the diseased and softened middle lobe resting on the temporal bone, combined also with the morbidly increased sensibility of the parts.

The second case is still more interesting and instructive. It seems to prove very strikingly the truth of the now generally admitted opinion, that the chief function of the cerebellum is to produce a combined and harmonious action of the several muscles called into movement for the attainment of a given end, as in locomotion, &c. It also, as well as the last, illustrates the apparent dependence of the intellectual faculties on a healthy condition of the gray cerebral matter. On examining the brain in this case, there was found a mass, presenting the characters of encephaloid carcinoma, incorporated with the anterior extremity of the left lobe of the cerebellum, of which it seemed a prolongation ; passing forward in inseparable connection with the pons, and following the emerging fibres of the corresponding crus cerebri, it terminated about an inch further (without penetrating the ventricle,) in the medullary substance of the middle lobe of the left hemisphere; the principal seat of the morbid change was in the commissural fibres which contribute to the formation of the pons. The medullary substance was alone diseased, the gray matter being distinct, and to all appearance healthy. The several nerves passing through the diseased mass were more or less vascular and soft, which would account for the various disturbances in function presented during life by the parts to which they were distributed, as deafness, &c.

The chief points in the symptoms of this disease to which attention need be directed in this notice are, that there was no paralysis of motion or sensation to the last, no convulsive movements, neither was one side of the body more distinctly implicated than the other, but the controlling, the co-ordinating power of the muscular system appeared abolished or nearly so ; thus, in the early part of the affection, the patient staggered during walking, as if slightly intoxicated, and was unable to direct her progress in a straignt line, she constantly deviating to the left ; as the disease advanced, the irregularity in the voluntary movements increased, and gradually affected the arms as well as the legs. She required to be held and directed in every act, supported at times by two assistants, and pushed forward by a third to enable her to move about the room ; and if by accident she fell when attempting any effort alone, she was quite unable to rise or to assist herself. She was perfectly sensible throughout, and the only change in her mental condition was a kind of restless excitement, with a certain indescribable feeling of distress, a childishness of thought with great feebleness of memory and attention ; often intervals of unexpected and remarkable revival of natural feelings and mental powers would occur, the cause of which is quite inexplicable.

A curious case is related by M. Blaquière, in which a ball discharged from a pistol struck a child, aged four years and a half, on one temple, traversed the brain, and passed out at the other temple; the child survived until the 29th day after the accident. When seen by M. Blaquiére upon the 20th, and several subsequent days, he was found sitting up in bed, amusing himself with his play-things, and eagerly calling for more food than was allowed him; he was lively and cheerful, except during the dressing of the wounds, manifesting the possession of all his mental faculties. After death it was found that the cranium had been perforated about an inch and a half above the external orbital process on each side; the anterior part of both cerebral hemispheres had been traversed by the ball; in front of the canal occasioned by its passage, there was a layer of cerebral substance six or eight lines in thickness, the gray matter above the canal was untouched, the ventricles of the brain were also uninjured.

21. *Reflex function of the Brain.* In a paper read before the last meeting of the British Association at York, Dr. Laycock offered further evidence confirmatory of his opinion published four years ago, that the brain, although the organ of consciousness, was subject also to the laws of reflex action, and that in this respect it did not differ from the other ganglia of the nervous system. He was led to this opinion by the general principle that the ganglia within the cranium being a continuation of the spinal cord must necessarily be regulated as to their reaction or external agencies by laws identical with those governing the functions of the spinal ganglia, and their analogies in the lower animals; and on investigation he found that observations and arguments like those satisfactorily adduced in proof of the existence of the reflux functions of the spinal ganglia, might be brought forward in proof that the cerebral ganglia have similar endowments.

He considers that the cerebral nerves, but especially the optic, auditory, and olfactory, are incident excitor nerves; that impressions made on them will pass on to the central axis, thence be communicated to the motor nerves, and thus give rise to combined muscular acts, or irregular and spasmodic movements. Similar acts may also have a centric origin, the exciting cause being *within* the brain. These acts may likewise be shown to be instinctive. In these particulars there is an evident analogy with the reflex functions of the spinal cord. To prove this, we must apply the natural stimulus to these nerves; thus light must impinge on the optic nerve, sound on the auditory nerve; pricking or tearing will have no effect in proving their reflex influence. Dr. Laycock mentions hydrophobia as presenting a good illustration of these cerebral reflex movements. The acknowledged excito-motory phenomenon of hydrophobia may be induced, 1st. Through the sensual nerves of touch, as by the contact of water with the surface of the head, hands, chest, the lips and pharynx. 2d. By a current of air impinging on the face or chest. These causes act undoubtedly on the incident nerves of the cord, as mentioned by Dr. Marshall Hall. But 3d. A bright surface, as a mirror; 4th. The sight of water; 5th. The sound of water dropping; 6th. The idea of water, as when suggested to the patient that he shall drink;—all most indubitably induce excito-motor phenomena, as decided and distinct as the first and second causes.

Some of the spasmodic movements as well as being involuntary, have a conservative object in view, as shown in the attempts to remove water when presented, the expelling it from the lips with a violent spasmodic jerk, &c.

These are the chief physiological points contained in Dr. Laycock's admirable paper; the remaining part of it is somewhat too abstruse and metaphysical to admit of further notice here, but the whole is well worthy of attentive perusal.

22. *Animal electricity.* The highly interesting electro-physiological researches of Professor Matteucci of Pisa have recently excited considerable attention; the following are the chief facts established by his experiments: 1st, Muscle is a better conductor of electricity than nerve, and nerve conducts better than brain; the conducting power of muscle may be taken as four times greater than that of brain or nerve.

2d. In the muscles of living animals, as well as those recently killed, an electric current exists, which is directed from the interior of each muscle to its surface. The duration of this muscular current corresponds with that of contractility: in cold-blooded animals, therefore, it is greatest; in mammalia and birds it is very brief. Temperature has a considerable influence on the intensity of the current, a small amount of electricity, being developed in a cold medium, a larger one when the medium is moderately warm. Any circumstance which enfeeble the frogs (the animal experimented on) and derange their general nutrition, will diminish the power of the muscles to generate electricity, as they also impair the contractile force. The muscular current appears to be quite independent of the nervous system. It is uninfluenced by narcotic poisons in moderate doses, but is destroyed by large doses, such as kill the animal. The development of this muscular current seems evidently to depend on the chemical action constantly taking place as an effect of the changes accompanying nutrition; these organic changes, in short, give rise to an electric current, just as do the chemical changes, attending the mutual reaction of inorganic materials, such as the reaction between a plate of metal, and an acidulated fluid in the ordinary voltaic pile. That considerable chemical changes attend the process of nutrition in muscle, seems evident when we consider the constant supply and waste of material of which it is the seat, and the evolution of sensible heat which accompanies its contraction; in this way the generation of electricity can be readily accounted for; the muscular fibre represents the metal acted on in the arrangement of the voltaic apparatus, and the arterial blood corresponds to the acidulated fluid. The surface of the muscle, which is more or less tendinous, and therefore different in structure and in function from the interior, represents the second plate of metal used in the voltaic apparatus, which does not suffer chemical action, but which only serves to form the circuit. The direction of the muscular current, therefore from the interior to the surface of the muscle is just such as might be expected, supposing it to be due to a chemical action taking place in the interior of the muscle.

3d. Another result obtained by M. Matteucci is the proof of the existence in frogs of an electric current distinct from the muscular current; it proceeds from the feet to head, and is peculiar to the Batrachian reptiles.

4th. Some curious results were obtained by applying electricity in various ways upon nerves. Upon making some experiments on the sciatic nerves of rabbits, he found that upon *closing* the circuit of the *direct* electric current, or the current directed from the brain to the nerves, contractions in the muscles of the posterior limbs were produced whilst upon *opening* this circuit marked signs of pain, with contraction of the muscles of the back, and feeble contractions of the posterior limbs, were caused. Upon *closing* the circuit of the *inverse* current, or that directed from the nerves to the brain, signs of pain, contractions of the muscles of the back, and feeble ones of the posterior limbs, were produced ; upon *opening* it, contractions of the posterior limbs were caused.

It will not be misplaced to notice here the marked analogy between the actions of the electrical organ of the torpedo and those of muscular fibre, which Matteucci's interesting experiments illustrate. Both are organized to act in a particular way; the one to develop electricity without any visible change in itself; the other to contract, with a demonstrable evolution of both heat and electricity. Both will manifest their peculiar phenomena by direct irritation, or by indirect irritation through the nerves. Both are brought under the control of the will by the nerve; the section of which paralyses the influence of the will over both, but does not destroy the peculiar power of either. In the electrical fish, irritation of the electrical lobe of the brain is capable of exciting a discharge of the organ, just as irritation of a segment of the spinal cord causes contraction of the muscles supplied by it. A current of electricity transmitted through the electrical organ or its nerves, causes discharge; and a similar current sent through a muscle of its nerves, causes it to contract. All the circumstances which modify the nutrition of muscle, will similarly affect that of the electrical organ.

23. *Nerves of the eighth pair.* Numerous observations have been lately made as to the respective functions of the glossopharyngeal, pneumogastric, and accessory nerves, by Stilling, Van Kempen, Bernard, and Hein. Their conclusions, on the whole, agree pretty closely, though they differ on some points, both with each other, and with previous experimenters. The present state of our knowledge (incomplete though it is) regarding the probable functions of these nerves, deducible from recent and previous facts, is thus stated by Mr. Paget.

1st. The glossopharyngeal is chiefly the nerve of the sense of taste, and in a less degree, a nerve of common sensation.

2d. The glossopharyngeal is, according to the experiments of Müller and Hein, the motor nerve of the stylopharyngeus, and probably also of the palatoglossus. Its branches to the digastricus, stylohyoideus, and constrictors of the pharynx, appear to be sensitive ones, or else derived from the facial and accessory nerves, with which it has previously united.

3d. The pneumogastric is, from its origin, composed of both sensitive and motor fibres. But it is undecided whether it alone supplies any particular muscles, or whether, in all its muscular branches, and especially in those given off above the œsophageal, there are filaments from the accessory as well as from its own roots.

4th. The accessory nerve contains, in all its lower roots, motor fibres alone ; in its upper roots, it is not improbable that there are some sensitive fibres also. It is a motor nerve of the sterno-mastoid and trapezius muscles; and very probably it gives, by this internal branch and other communications, motor fibres to the pneumogastric, from which they are subsequently distributed to some or all of the muscles of the larynx and pharynx ; and, in some animals, to the muscles of the palate.

The main difficulty in assigning exactly to the pneumogastric and spinal accessory nerves their respective functions, depends on the intimate commingling of the fibres forming the uppermost roots of the accessory with those forming the lower roots of the pneumogastric ; so that it is hard to say whether some fibres belong to the one nerve or to the other; therefore as Mr. Paget observes, before we can hope to distinguish precisely the physiological properties of these two nerves, we must learn to distinguish them (if, indeed, they are two nerves,) anatomically.

24. *Anterior thoracic nerve.* Dr. Hargrave considers that the anterior thoracic nerve from the brachial plexus, inasmuch as it supplies the subclavius, pectoralis major and minor muscles, which muscles, he says, are especially concerned in dyspnea and orthopnea, ought to be added to the respiratory system of nerves, as laid down by Sir C. Bell. This nerve, he states, performs a function analogous to that performed by the external inferior respiratory nerve in respiration, namely, to associate in this movement, the muscles to which it is distributed, with the serratus magnus, the diaphragm, sternomastoid, and trapezius muscles. Dr. Hargrave, therefore, proposes to call this nerve the *anterior*, *inferior*, *external respiratory*, in reference to the aspect of the thorax to which it is distributed.

25. *Pacinian corpuscles.* The following account of these little bodies is given by Mr. Paget. " The investigations of Henle and Kölliker have proved a new and peculiar mode of peripheral termination of the nerve fibres in the little bodies, seated especially in the nerves of the fingers and toes, which were discovered, and to a certain point well described, by Pacini, of Padua, in 1830. These *Pacinian corpuscles* are found in man at all ages after the twenty-second week of fœtal life, and under all circumstances, and in many mammalia. They are most numerous on the cutaneous nerves of the hands and feet; but they occur also, sometimes, on other sensitive cerebro-spinal nerves, and on the sympathetic plexuses in the mesentery and mesocolon, and about the pancreas, where they are especially numerous in cats. In man, from 150 to 350 may be counted on a single limb; and they are chiefly abundant on the branches of the digital nerves, just penetrating the cutis, to which they are attached singly or in pairs, or sometimes in groups, by little fibro-cellular pedicles. Through the pedicle of each, a single primitive nerve-fibril passes into the corpuscle. The corpuscles are of various forms, elliptic, ovate, obovate, crescentic, or reniform; they measure (in parts of a line) from ·66 to 1·2 in length, and from ·45 to ·6 in breadth. They are semi-transparent, slightly glistening, and appear as if a central cord passed through them. Each of them is composed of from 40 to 60 very thin coats, arranged round a central canal or cavity, like so many capsules inclosed one within another; and each coat or capsule is composed of two layers of fibro-cellular tissue, an outer layer with circular, and an inner with longitudinal fibres. Between each two adjacent layers or capsules, there is an albuminous fluid; it is most abundant between the outer capsules, which are less compactly arranged than the central ones. The outermost of all the capsules in each corpuscle is connected by cellular tissue with the adjacent parts, from which also blood vessels penetrate inwards through more than half the layers. Here and there the adjacent capsules appear connected by partial septa extending across the spaces containing the fluid, and this is especially the case at the end opposite the pedicle. The canal or cavity in the axis of each corpuscle contains a fluid like that between the capsules, and, in this fluid, a primitive nerve-fibril. The nerve-fibril, after traversing the pedicle of the corpuscle, and a conical prolongation from the end of the pedicle through the substance of the lower part of the corpuscle, enters the cavity, and at once becomes smaller, paler, and flatter. It passes along the cavity, and at or near its distal end, terminates in a knob, or by bifurcating; in no case is anything formed like the *terminal loops* of nerves, and it is very rarely that more than one nervous fibril enters a corpuscle; neither does the terminal enlargement of the nerve-fibril resemble a ganglion corpuscle. Of the use of these bodies little can be said. It is suggested, that as their construction with alternate layers of membrane and fluid is rather like that of the electric organs of the electric ray, &c., these also may be electric organs, and, according to Pacini, the chief agents in mesmeric operations. But Henle and Kölliker could find no manifestations of free electricity in them during life. Their not occurring upon any known motor nerves, would appear to prove that they have nothing to do with motion; but their existence on many nerves of the sympathetic system, and their non-existence on many sensitive nerves make it probable that they are not connected with acuteness of sensation. They may be electric organs, as their peculiar form suggests, but before they can be concluded to have any relation to animal magnetism, it would be advisable to prove that *that* has any relation (except in name) to physical magnetism, or any form of electricity."

26. *Sympathetic system: use of the sympathetic nerve and its ganglia.* With regard to the function of the sympathetic system, Dr. Procter says: "The nearest approach to a positive determination of its use that we can come to with our present limited knowledge is, that it is for the purpose of regulating the tonic contraction of the arterial system, and for *nothing else.*" He states the necessity of so important a system as the arterial, having a controlling and directing power, and observes, "that in all parts of the animal body, where large and sudden supplies of blood are required at irregular periods—such as the heart, stomach, intestines, and organs of generation; there we have the ganglionic or sympathetic system very fully developed.' He explains the reason why so few nerves of the sympathetic system are found accompanying the arteries of the extremities, by the fact that the parts to which these vessels are distributed do not require the same large, sudden, and irregular supplies of blood as do the several organs and viscera of the body—[and yet the condition of a limb in full action compared with its condition after a long rest, differs as much in regard to the quantity of blood circulating through it, as does that of an organ in full secretion compared with that of the same organ in a quiescent state, so that Dr. Procter's reason for the comparative absence of sympathetic fibres accompanying the arteries of the limbs, does not explain the circumstance sufficiently.]

27. *Splanchnic nerve.* M. Bourgery, in a late memoir, considers that the splanchnic system of nerves consists of five parts. 1st. Of some visceral and organic nerves, the fundamental part of the splanchnic nervous system. 2d. Of ganglionic portions, which are considered as the general centres of excitement and of harmonization of that group of organs, and usually, as functional auxiliaries to each other. 3d. Of *extra-visceral* plexuses, or chains of communication between the different organs of the same group, between these and the various ganglionic centres, and between these various centres themselves. 4th. Of the two longitudinal chains of communication with the central extremity of the nerves, or properly the two cords parallel with the cerebro-spinal axis, known under the name of the great sympathetic. 5th. The last part of the splanchnic nervous system consists of anastomoses of the ganglionic nerves with the peripheral extremities of the cerebro-spinal nerves. M. Bourgery also mentions having observed in great abundance the nerves of synovial and serous membranes, and states also that he has observed ganglia and gray nervous matter on certain parts of the cerebro-spinal nerves, especially the trigemini and pneumogastric nerves, which offer some explanation for the similarity in function between these nerves and the great sympathetic observed by physiologists.

28. *Union of divided extremities of nerves.* The possibility of the divided extremities of two nerves of totally different functions being made to unite with each other has been again advanced by M. Tavignot. The conclusions he has arrived at coincide with the observations previously made by M. Flourens, though they are opposed to the results obtained by Dr. Bidder, who experimented on the lingual and hypoglossal nerves, and was led to the conclusion that such an union does not take place. M. Tavignot considered that since any nerve which has been divided may have its continuity and its functions completely restored by keeping its divided extremities in apposition for some time, so also it might be possible that the divided extremities of nerves of different functions might be induced to unite, and that the function of each nerve might thus be restored. From some experiments which he made on this subject he found:—1st. That if two adjoining nervous cords of different functions be included together in a single ligature so as to effect their simultaneous division, there is shortly developed between the four extremities a kind of nervous ganglion which is common to each of them, and in which the fibres of the two nerves and their functions seem confounded; and 2d. That if two adjoining nerves of different functions be divided, and the upper end of one be adapted to the lower end of the other and kept in apposition, the formation of a new nerve preserving the functions of the old one is effected. [The latter of these conclusions is so opposed to the results of Dr. Bidder's experiments, and so far from being in conformity with the laws by which the functions of the nervous system seem governed, that further observations are requisite before its probability can be admitted.]

29. *Re-establishment of sensibility in autoplastic flaps.* Some curious observations have been offered by M. Jobert de Lamballe, which seem to show that the sensibility of flaps in autoplastic operations is restored through the medium of blood-vessels, and not through that of demonstrable nerve-fibres. The following are his observations: 1st. Immediately after autoplastic operations the sensibility of the flap diminishes or disappears: this is in direct relation with the loss of blood. 2d. Until the section of the pedicle some degree of sensibility is retained. 3d. At the expiration of a certain period after this section, vascularity and sensibility re-appear in the flap simultaneously, and increase in an equal ratio. 4th. In many cases the vascularity of the flap becomes considerable, and its sensibility is then increased in a proportional degree. Anatomical investigation has furnished the following facts:—1st. The autoplastic flaps after the section of the pedicle, are isolated from the rest of the system by a cicatrical tissue. 2d. There exists as means of communication between the flaps and the rest of the organization, only those vessels which traverse the tissues of the cicatrix; nervous filaments are never seen in this new formation. 3d. The nerves which originally existed in the flap, waste and eventually disappear. 4th. The nerves of the part surrounding the flap stop at its borders; sometimes they terminate abruptly in a kind of enlargement of the neurilema, at others they are lost in the tissue of the cicatrix, without its ever being possible to trace them into the flap.

30. *Functions of the nervous system.* Some curious views regarding the functions of the nervous system have recently been advanced by Natansen. He considers that each nerve of sensation is composed of several kinds of nerves, each of which has its own peculiar function. Thus the nerves of touch would comprise those which perceive temperature, others which perceive the resistance of bodies, and lastly, others possessing the property of touch, properly so called. In proof, Natansen instances the fact, that either of these faculties may be temporarily lost without the other two being so ; thus, when the arm has been "asleep," and sensibility is returning in it, the hand first perceives temperature, then the resistance of bodies, and it is only after some time that the faculty of touch properly so called can be exercised ; in the lower extremities the contrary takes place, the sense of touch first returns, then we experience a sensation of pricking followed by the perception of temperature, whilst the faculty of appreciating the resistance of bodies returns last. With regard to the optic nerve, he admits three kinds of nerves: those of red, of blue, and of yellow light, which are the bases of all other colours. It is not alone the optic nerves which admit of being impressed by light—light may also act upon the nerves of the eyelids, not so as to produce the phenomena of vision, but so as to excite a sense of pricking; in proof of this M. Natansen states, that he has often observed in blind individuals, even where the globe of the eye has been lost, that light has occasioned to them a painful sensation almost amounting to "photophoby." He analyses in the same manner the senses of taste, of touch, &c., and considers that they all confirm his statement, that all nerves of sensation are not simple, but are composed of several different kinds of nerves,to each of which belongs a peculiar function different to that of the others. He ventures also to explain the varieties of the intellectual faculties in the same way.

31. *Relative weight of the different portions of the brain.* M. Bourgery finds that the mean weight of the encephalon, or central nervous mass being 20393·5 grains troy,the cerebral hemispheres stand for 16940·46 grains of that quantity, the cerebellum for 2176·7 grs., the cephalic prolongation of the cerebro-spinal axis for 1312·2 grs., of which the optic thalami and compora striata take 879·9 grs. ; the medulla oblongata, with the pons varolii, 432·2 grs., and the spinal cord 710·1 grain. Hence, in man, the cerebral hemispheres include a nervous mass which is *four* times that of all the rest of the cerebro-spinal mass, *nine* times that of the cerebellum, *thirteen* times that of the cephalic stem of the spinal cord, and *twenty-four* times that of the spinal cord itself.

32. *Eye. Action of the oblique muscles.* Dr. George Johnson has performed some experiments to determine the action of the oblique muscles of the eye, and has obtained results similar to those arrived at by Volkman and others, proving the truth of Hunter's opinion, that these muscles rotate the eyeball on its antero-posterior axis, and so keep the eye steadily fixed on an object we are regarding, during certain movements of the head, as from shoulder to shoulder, (the effects of which are not corrected by the recti muscles,) and thus enable the image of the object to be kept on the same point of the retina,and not be allowed to move over its surface, which it would do, during these movements of the head, were there no oblique muscles to counteract this tendency. In Dr. Johnson's experiments, a dog was killed by the injection of air into a vein, and immediately the inferior oblique muscle was exposed by dissecting off the conjunctiva without in any way interfering with the surrounding parts ; by means of two fine wires, a slight electric current was then directed through the muscle. The effect was a rapid rotation of the eye upon its antero-posterior axis, so that a piece of paper placed at the outer margin of the cornea passed downwards and then inwards towards the nose. The superior oblique was then exposed at the back of the orbit, and was treated in the same manner. The rotary movement produced was precisely the reverse of the former; the paper at the outer margin of the cornea passed upwards, and then inwards towards the nose. In the case of the superior oblique the movement was less extensive, the irritability of the muscle being less, perhaps from the delay in exposing it and from some slight injury inflicted on it in so doing. There could be no doubt as to the direction of the movement in both cases; there was not the slightest appearance of elevation, depression, abduction, or adduction, of the cornea. The experiment was subsequently repeated on another dog with precisely the same result.

33. *Muscæ volitantes.* Dr. Jago considers that those minute globular particles, which may always be seen in the healthy eye by looking through a card with a small aperture, (and which, when in great abundance, and visible without looking through the aperture in a card, are called muscæ volitantes,) are seated in the vitreous humour, and constitute a natural and essential part of this fluid. That they exist in a fluid is manifest from their floating and moving about in the interior of the eye; thus, when we raise the eye to look at an object, they evidently continue to move in the same direction even after the eye is stopped; and then, after balancing a moment, commence to descend again to their usual places. That they are near to the retina and far from the cornea seems proved by this experiment; when a card (through a small aperture in which we are looking) is moved across the axis of the eye in any direction, these little particles move in the same direction, but through a less space than that travelled by the card, or by a tear which we can see. These two circumstances seem to point to the vitreous humour as the seat of muscæ volitantes.—*Half Yearly Abstract.*

(To be Continued.)

